# Matrix metalloproteinase promotes elastic fiber degradation in ligamentum flavum degeneration

**DOI:** 10.1371/journal.pone.0200872

**Published:** 2018-08-01

**Authors:** Kazuki Sugimoto, Takayuki Nakamura, Takuya Tokunaga, Yusuke Uehara, Tatsuya Okada, Takuya Taniwaki, Toru Fujimoto, Hiroshi Mizuta

**Affiliations:** Department of Orthopedic Surgery, Faculty of Life Sciences, Kumamoto University, Chuo-ku, Kumamoto, Japan; University of Louisville, UNITED STATES

## Abstract

Ligamentum flavum (LF) hypertrophy in lumbar spinal canal stenosis (LSCS) is characterized by a loss of elastic fibers and fibrosis. Chronic inflammation is thought to be responsible for the histological change but the mechanism underlying elastic fiber degradation remains unclear. Given that matrix metalloproteinase (MMP)-2 and -9 have elastolytic activity and are partly regulated by inflammatory cytokines such as interleukin (IL)-6, in this study, we investigated whether MMPs mediate LF degeneration using 52 LF samples obtained during lumbar surgery, including 31 LSCS and 21 control specimens. We confirmed by histological analysis that the LSCS samples exhibited severe degenerative changes compared with the controls. We found that MMP-2 was upregulated in LF tissue from patients with LSCS at the mRNA and protein levels, whereas MMP-9 expression did not differ between the two groups. The MMP-2 level was positively correlated with LF thickness and negatively correlated with the area occupied by elastic fibers. *IL-6* mRNA expression was also increased in LF tissue from patients with LSCS and positively correlated with that of *MMP-2*. Signal transducer and activator of transcription (STAT)3, a component of the IL-6 signaling pathway, was activated in hypertrophied LF tissues. Our *in vitro* experiments using fibroblasts from LF tissue revealed that IL-6 increased MMP-2 expression, secretion, and activation via induction of STAT3 signaling, and this effect was reversed by STAT3 inhibitor treatment. Moreover, elastin degradation was promoted by IL-6 stimulation in LF fibroblast culture medium. These results indicate that MMP-2 induction by IL-6/STAT3 signaling in LF fibroblasts can degrade elastic fibers, leading to LF degeneration in LSCS.

## Introduction

Lumbar spinal canal stenosis (LSCS) is a disease associated with locomotor dysfunction in elderly people; the number of patients with LSCS is expected to increase with the aging of the population [1, 2]. Narrowing of the spinal canal in LSCS causes lower back and leg pain, numbness, and intermittent claudication through direct nerve compression; hypertrophy of the ligamentum flavum (LF) is the primary cause of LSCS [3, 4]. Normal LF is composed of 70% elastic and 30% collagen fiber, but degenerative LFs exhibit a reduction and fragmentation of elastic fibers and an excess of collagen fibers, resulting in LF fibrosis and hypertrophy [5, 6]. Furthermore, the loss of LF elasticity may cause LF folding within the spinal canal, thereby aggravating spinal canal narrowing [6].

Among the factors contributing to LF degeneration, such as aging, mechanical stress, and genetics, repeated inflammation caused by mechanical stress-induced tissue damage is thought to stimulate the repair process in LFs and subsequent hypertrophy [7–9]. We previously showed that angiopoietin-like protein (Angptl)2, a mediator of chronic inflammation, is highly expressed in LF tissues of patients with LSCS and is induced in LF fibroblasts by mechanical stress; furthermore, Angptl2 stimulates transforming growth factor -β1 expression, leading to LF fibrosis, and interleukin (IL)-6 expression [10, 11]. IL-6 is an important cytokine involved in acute and chronic inflammation that actively influences extracellular matrix (ECM) remodeling in various diseases [12, 13].

Some studies have reported that matrix metalloproteinases (MMPs) may be responsible for degrading the elastic fibers in LF tissues [14, 15]; however, the detailed molecular mechanism remains unclear. MMPs are critical factors in normal physiological processes such as ECM remodeling and are implicated in inflammatory disorders such as arthritis, lumbar disc herniation (LDH), and cardiovascular disease [16, 17]. MMP-2 and -9 are gelatinases that have elastolytic activity and are regulated by inflammation, including inflammatory molecules such as IL-6 [13, 18, 19].

In this study, we show that MMPs are responsible for LF degeneration in patients with LSCS and that IL-6 promotes MMP-mediated elastic fiber degradation. Our findings provide new insight into the etiology of LSCS and suggest that MMPs are potential therapeutic targets for disease treatment.

## Methods

### Patients

The Kumamoto University Hospital Ethics Committee approved this research (no. 1303), and informed consent was obtained from all patients. This study was performed in accordance with the Declaration of Helsinki (1975).

In total, LF samples were obtained for this study from 52 patients (28 male and 24 female) who underwent lumbar surgery with removal of LF tissue at Kumamoto University Hospital from July 2015 to August 2017. The thickness of the LF was quantified at the facet joint level by magnetic resonance imaging (MRI) [10, 11]. The maximum size of the LF was measured twice and the mean value was taken as the sample thickness. 31 LF specimens with MRI-confirmed LSCS comprised the LSCS group (mean age: 71.4 years; range: 52–92 years; 17 males and 14 females), whereas 21 LFs from patients with lumbar diseases other than LSCS such as LDH and cauda equina tumor comprised the control group (mean age: 52.8 years; range: 23–85 years; 11 males and 10 females). None of the patients had previously undergone lumbar surgery.

### Histology

Harvested LF tissues were fixed in 4% paraformaldehyde (PFA), embedded in paraffin, and cut into 4-μm-thick sections. Hematoxylin-eosin (HE) staining and picrosirius staining were performed according to standard procedures. To evaluate ECM degeneration in LF tissues, the sections were stained using Trichrome Stain kit (Modified Masson’s) (ScyTek Laboratories Inc., Utah, USA) for collagen evaluation, Elastica-van Gieson (EVG) staining kit (Abcam, Cambridge, MA, USA) for quantitative analysis of elastic fibers and Weigert’s resorcin-fuchsin stain (Nacalai Tesque, Kyoto, Japan) with or without peracetic acid oxidation for analysis of the elastic system morphology (oxytalan, elaunin, and elastic fibrils) [20]. Picrosirius-stained images were acquired via polarization microscopy (Olympus BX-51; Olympus, Tokyo, Japan) and other images were taken on a BZ-X700 microscope (Keyence, Osaka, Japan). The severity of LF fibrosis and elastic fiber degradation was graded using the scoring systems of Park et al. [14]. For fibrotic scoring, normal tissue has no fibrotic region (grade 0), whereas fibrosis in 25% or less, between 25% and 50%, between 50% and 75%, and over 75% of the entire area was scored as grade 1, 2, 3, and 4 respectively. Similar to fibrosis evaluation, for elastic fiber scoring, normal tissue shows no elastic fiber degradation (grade 0), whereas the degradation in 25% or less, between 25% and 50%, between 50% and 75%, and over 75% of the entire area was scored as grade 1, 2, 3, and 4 respectively. The number of fibroblasts and the occupation ratio of collagen fibrils or elastic fibers were calculated using the BZ-X Analyzer (Keyence). Regions of interest were randomly selected from 18 sites in each LF (nine sites each on the dural and dorsal sides). Additionally, for analysis of the chondrometaplasia accompanied by LF degenerative changes [5, 15], after homogenization of 1 mg of LF tissue and extraction using guanidine hydrochloride, the proteoglycan levels in LF tissues were examined using a proteoglycan detection kit (ASTARTE, Bothell, WA, USA) according to the manufacturer’s instructions.

### Reverse-transcription polymerase chain reaction (RT-PCR)

Harvested LF tissues were frozen in liquid nitrogen and stored at −80 °C until RNA extraction. The tissue was crushed using a Multi-bead Shocker (MB400U; Yasui Kikai, Osaka, Japan), and total RNA was extracted using TRIzol reagent (Invitrogen/Life Technologies, Carlsbad, CA, USA) and purified using the PureLink RNA Mini kit (Invitrogen). cDNA was synthesized from the RNA using PrimeScript RT Master Mix (Takara Bio, Otsu, Japan), followed by RT-PCR on an Applied Biosystems 7500 Fast Real-Time PCR system (Invitrogen) using SYBR Premix Ex Taq II (Takara Bio). Relative expression was calculated with the ΔΔCt method, with the *18S ribosomal RNA* (*18S rRNA*) gene used as an internal control. Primers used for RT-PCR of *MMP-2* and *-9*, *elastin*, *IL-6*, and *18S rRNA* are listed in Table 1.

**Table 1:** Sequences of primers used for RT-PCR analysis.

Gene	Sequences (5'-3')
*MMP-2*	
Forward	TTAAGCTTCCACTCCGGGCAGGATT
Reverse	GCGGATCCAGCGCCCAGAGAGACAC
*MMP-9*	
Forward	AGAGATGCGTGGAGAGTCGAA
Reverse	AAGGTTTGGAATCTGCCCAGG
*elastin*	
Forward	GGTGCGGTGGTTCCTCAGCCTGG
Reverse	GGGCCTTGAGATACCCCAGTG
*IL-6*	
Forward	TGTCCTGCAGCCACTGGTTC
Reverse	AAGCCAGAGCTGTGCAGATGAGTA
*18S rRNA*	
Forward	TTTGCGAGTACTCAACACCAACATC
Reverse	GAGCATATCTTCGGCCCACAC

MMP-2/-9, matrix metalloproteinase-2 and -9; IL-6, interleukin-6; 18S rRNA, 18S ribosomal RNA.

### Quantitative analysis of MMPs

Frozen LF tissue samples were homogenized and total proteins were extracted with the T-PER Tissue Protein Extraction Reagent (Thermo Fisher Scientific, Waltham, MA, USA). The extract was centrifuged for 15 min at 15,000 rpm (CF16RN; Hitachi Koki Co., Ibaraki, Japan) and 4 °C, and the protein concentration in the supernatant was determined with the Bradford method using a protein assay kit (Takara Bio). Total MMP-2 and -9 concentrations in each sample were measured with specific enzyme-linked immunosorbent assay (ELISA) kits (R&D Systems, Minneapolis, MN, USA).

### Immunohistochemical analyses

Following the same process as histopathological samples, 4-μm-thick sections of LF tissues were prepared. After pretreatment of the sections with Target Retrieval Solution (pH 9) (Dako, Glostrup, Denmark) in an autoclave (121 °C), endogenous peroxidase was blocked by treatment with 3% hydrogen peroxide in methanol for 20 min, and non-specific antibody binding sites were blocked with 10% goat serum (Nichirei, Tokyo, Japan). The following primary antibodies were used: anti-MMP-2 (mouse monoclonal, 1:200, AB86607), anti-MMP-9 (rabbit polyclonal, 1:200, AB38898), anti-phosphorylated extracellular signal-regulated kinase (p-ERK)1/2 (rabbit polyclonal, 1:200, AB214362), anti-phosphorylated (p-)p38 (rabbit polyclonal, 1:100, AB4822), and anti-phosphorylated c-Jun N-terminal kinase (p-JNK) (rabbit polyclonal, 1:100, AB4821) (all from Abcam), and anti-phosphorylated signal transducer and activator of transcription (p-STAT3) (rabbit polyclonal, 1:200, SC-7993; Santa Cruz Biotechnology, Santa Cruz, CA, USA). After treatment with a peroxidase-labeled secondary antibody (Histofine Simple Stain Max PO; Nichirei), immunoreactivity was visualized using Histofine 3,3'-diaminobenzine (Dojindo Molecular Technologies, Kumamoto, Japan) and nuclei were counterstained with hematoxylin. Images were acquired on a BZ-X700 microscope. For double immunofluorescence labeling, antibodies against MMP-2 (1:200) and IL-6 (rabbit polyclonal, 1:400, AB6672; Abcam) were used as the primary antibodies and Alexa Fluor 488-labeled anti-mouse IgG (1:500, R37120) and Alexa Fluor 594-labeled anti-rabbit IgG (1:500, R37117) (both from Invitrogen) were used as secondary antibodies. Nuclei were counterstained with 4',6-diamidino-2-phenylindole (DAPI).

### Isolation and culture of LF fibroblasts

LF tissue samples were harvested, minced, washed in physiological saline, and incubated for 1 h at 37 °C in Dulbecco’s Modified Eagle Medium (DMEM; Gibco, Grand Island, NY, USA) containing 0.2% type I collagenase (Gibco) and 1% penicillin-streptomycin (Gibco). This suspension was filtered using a 100-μm mesh cell strainer (BD Biosciences, Franklin Lakes, NJ, USA); the cells were then seeded in a 6-well plate (BD Biosciences) filled with DMEM containing 10% fetal bovine serum (Gibco) and 1% penicillin-streptomycin, and cultured at 37 °C in a 5% CO_2_ humidified incubator. The culture medium was changed twice a week. Subsequent experiments were performed using cells from primary cultures up to the third passage.

### Stimulation of LF fibroblasts with IL-6

To analyze the biological response of human LF fibroblasts to an inflammatory cytokine, 300 ng/ml recombinant IL-6 protein (Wako Pure Chemical Industries, Osaka, Japan) with or without the same amount of soluble IL-6 receptor α (sIL-6Rα; Wako Pure Chemical Industries) was added to a 12-well plate (BD Biosciences) containing subconfluent LF fibroblasts, followed by incubation for 12 h. LF fibroblasts without IL-6 stimulation served as a control. After the procedure, RNA was extracted from the cells and the expression of *MMP-2* and *-9* was evaluated by RT-PCR (the relative abundance of the target transcripts was normalized to the expression of *18S rRNA*). IL-6 was applied at concentrations of 0, 100, 300, and 1000 ng/ml, and the response was evaluated in the same manner. Additionally, the fibroblasts were stimulated for 6, 12, and 24 h in an incubator (37 °C, 5% CO_2_) and then analyzed in the same manner. To evaluate the effect of IL-6 stimulation on MMP protein expression, 300 ng/ml IL-6 was added to subconfluent LF fibroblasts cultured in a 6-well plate (BD Biosciences), which was then incubated for 24 h (37 °C, 5% CO_2_). The MMP protein level in the medium was analyzed by ELISA. A plate pretreated with 50 nM STAT-3 inhibitor V (Stattic; Cayman Chemical, Ann Arbor, MI, USA; CAS 19983-44-9) was also stimulated with IL-6 for 12 or 24 h for evaluation of mRNA and protein levels, respectively.

LF fibroblasts were seeded in culture slides (BD Biosciences) for immunofluorescence analysis. After IL-6 stimulation (300 ng/ml) and incubation for 30 min at 37 °C and 5% CO_2_, the cells were fixed in 4% PFA, blocked with 10% normal goat serum, and then incubated overnight at 4 °C with anti-p-STAT3 monoclonal antibody (1:200). Alexa Fluor 488-labeled anti-rabbit IgG (1:500, A27034; Invitrogen) was used as a secondary antibody and nuclei were stained with DAPI.

### Western blot analysis

To analyze the IL-6-induced phosphorylation of transcription factors, LF fibroblasts grown in a 6-well plate were incubated for 15 min with IL-6 (300 ng/ml) and lysed in radioimmmunoprecipitation assay buffer (Nacalai Tesque). The lysates were homogenized in sample buffer solution containing 2-mercaptoethanol (2×) (Nacalai Tesque) and proteins were separated by sodium dodecyl sulfate-polyacrylamide gel electrophoresis (SDS-PAGE) and transferred to a nitrocellulose membrane (Invitrogen) that was blocked in blocking reagent (Block-Ace; DS Pharma Biomedical, Osaka, Japan) for 30 min at 25 °C and treated overnight at 4 °C with the following primary antibodies: anti-human STAT3 (mouse monoclonal, 1:500, SC-8019) and p-STAT3 (1:500) (both from Santa Cruz Biotechnology); and anti-human ERK1/2 (mouse monoclonal, 1:1000, AB54230), p-ERK1/2 (1:1000), p38 (rabbit monoclonal, 1:1000, AB170099), p-p38 (1:1000), JNK (rabbit polyclonal, 1:500, AB112501), and p-JNK (1:500) (all from Abcam). The membrane was then incubated with horseradish peroxidase-conjugated goat anti-mouse IgG (Santa Cruz Biotechnology) for 1 h at 25 °C and immunoreactivity was visualized with Chemi-Lumi One Super (Nacalai Tesque) and a luminescent image analyzer (EZ-capture2; ATTO, Tokyo, Japan) and quantified using ImageJ v.1.47 software (National Institutes of Health, Bethesda, MD, USA).

### Quantitative analysis of MMP activity

LF fibroblasts with or without 50 nM Stattic pretreatment were stimulated with IL-6 (300 ng/ml) for 24 h. MMP-2 and -9 activities in the culture supernatant were measured with an SDS-PAGE gelatin zymography kit (Cosmo Bio, Tokyo, Japan) according to the manufacturer’s instructions. MMP-2 and -9 activities were evaluated by densitometric analysis of the bands using a flatbed photo scanner (ES-10000G; Epson, Nagano, Japan) and ImageJ v.1.47 software. Next, fibroblasts cultured in a 6-well plate in the presence or absence of Stattic were stimulated with IL-6 for 24 h; 1 mg of soluble elastin protein (elastin from bovine neck ligament; Sigma-Aldrich, St. Louis, MO, USA) was added to the medium for 6 h, and the concentration of soluble elastin in the supernatant was measured using a competitive ELISA kit (SK00806-01; Aviscera Bioscience, Santa Clara, CA, USA) according to the manufacturer’s instructions.

### Statistical analysis

Data are expressed as the mean ± the standard error of the mean (SEM). Student’s t test was used to assess differences between two groups, with P < 0.05 considered statistically significant.

## Results

### Pathological changes in LSCS samples

**Fig 1:**
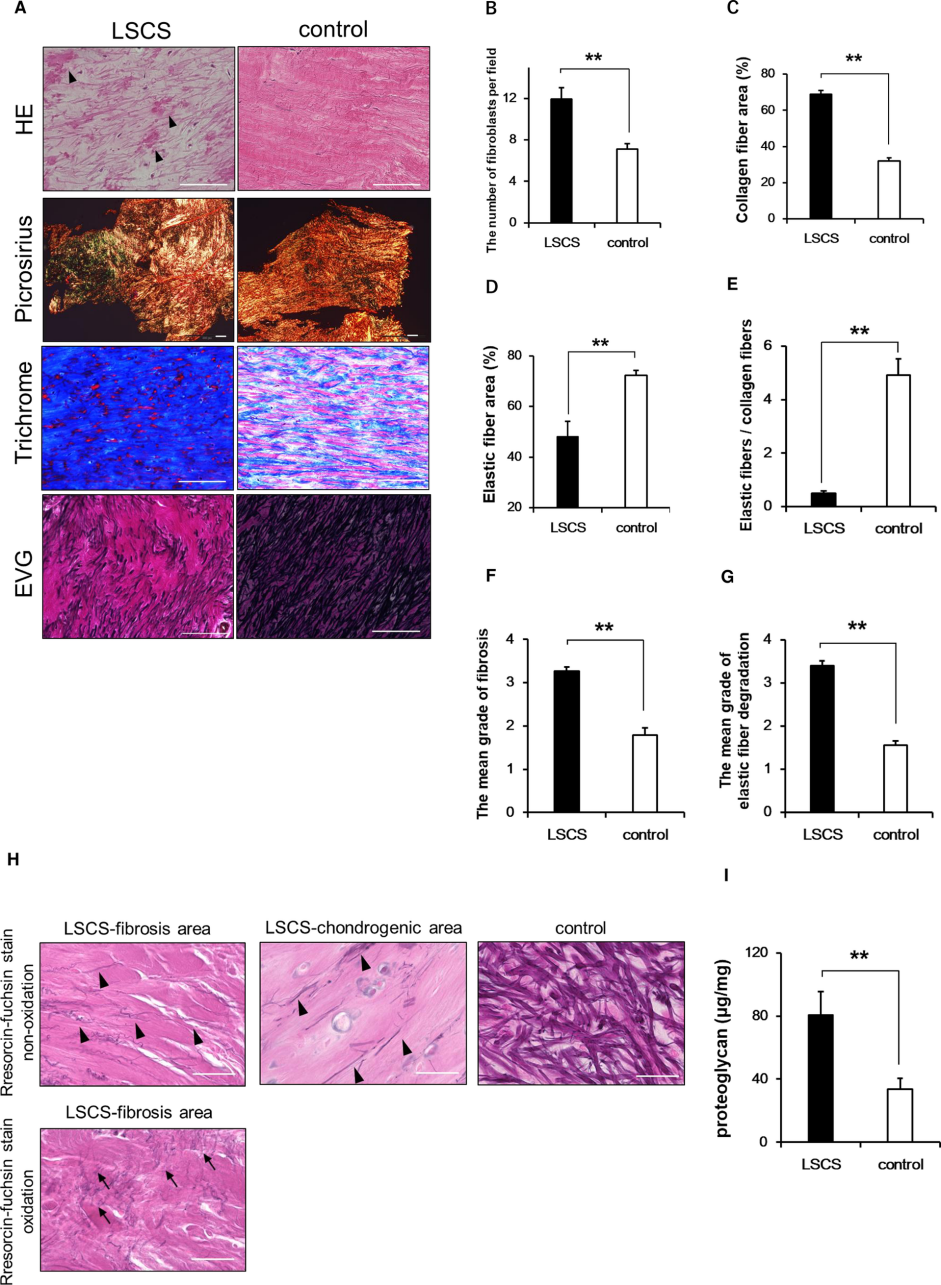
Pathological changes in LF tissue. **A:** Hematoxylin-eosin (HE), picrosirius, trichrome, and Elastica-van Gieson (EVG) staining of LF in LSCS and control samples. Arrowheads indicate hyaline degeneration. Type 1 collagen appears red or orange and type 3 collagen appears green in color. Each scale bar, 100 μm. **B:** Average number of fibroblasts in the randomly selected field (×400). **C, D:** Average occupation of collagen fibers (C) and elastic fibers (D). **E:** Ratio of elastic and collagen fibers. **F, G:** Mean grade of LF fibrosis (F) and elastin degradation (G) by Park’s scoring systems. Lower grade (0 and 1) indicates less degeneration and grade 4 indicates severe degenerative change. **H:** Resorcin-fuchsin staining of LF samples in LSCS-fibrosis, LSCS-chondrogenic, and the representative control area. Arrowheads indicate elaunin-like fibers and arrows indicate oxytalan-like microfibers. Scale bar, 50 μm. **I:** Quantitative proteoglycan levels of LF tissues in LSCS and control. Data represent the mean ± SEM (n = 13 per group for histological assay; and n = 20 per group for proteoglycan assay, selected randomly). **P < 0.01 vs. control group.

The LF was thicker in the LSCS than in the control group (Table 2). Spindle-shaped fibroblasts were randomly distributed and ran in parallel with the regularly arranged collagen and elastic fibers in control LF samples, whereas fibroblasts with enlarged nuclei were detected, and the elastic fibers were notably decreased and disintegrated with irregularly distributed collagen fibers in LSCS samples on HE staining (Fig 1A). Additionally, the hyaline degeneration regions were scattered and the number of fibroblasts was increased in the samples from the LSCS patients (Fig 1B). Picrosirius staining showed the areas with high expression of type 3 collagen (green) as well as type 1 collagen (red or orange) in samples from the patient group, whereas type 3 collagen expression was not observed in controls (Fig 1A), indicating LSCS-LFs activated fibrotic response [21]. Hypertrophied LFs from the LSCS patients exhibited increased collagen fibers (blue area) and decreased elastic fibers (black area) quantitatively on trichrome and EVG images (Fig 1C and 1D). The ratio of elastic and collagen fibers was significantly decreased in LSCS-LFs relative to controls (Fig 1E). The mean grade of fibrosis and elastic fiber disruption of the LF was significantly higher in the LSCS group than in the control group (Fig 1F and 1G). Regarding the elastic system morphology, most of the control samples were made up of thick elastic fibers separated by a small collagen area, but the elaunin-like thin fibers were detected at the strongly fibrotic or chondrogenic areas in LSCS samples on Weigert’s resorcin-fuchsin stain without oxidation. Moreover, after oxidation, oxytalan-like microfibers branching from the elaunin-like fibers were found in the LSCS samples (Fig 1H). The proteoglycan levels of the LSCS group were significantly increased relative to the control group (Fig 1I). These findings indicate that our patient samples showed degenerative changes compared to the controls.

**Table 2:** Demographic data of the study population.

	LSCS group	Control group	P-value
Number of patients	31	21	
L1/2	0	2	
L2/3	5	4	
L3/4	8	3	
L4/5	17	8	
L5/S1	1	4	
Age	71.4±1.5	52.8±4.3	<0.01
Sex	17/14	11/10	0.768
LF thickness	5.3±0.2	3.3±0.1	<0.01

LSCS, lumbar spinal canal stenosis; LF, ligamentum flavum.

### MMP-2 and -9 expressions in LF tissue

**Fig 2:**
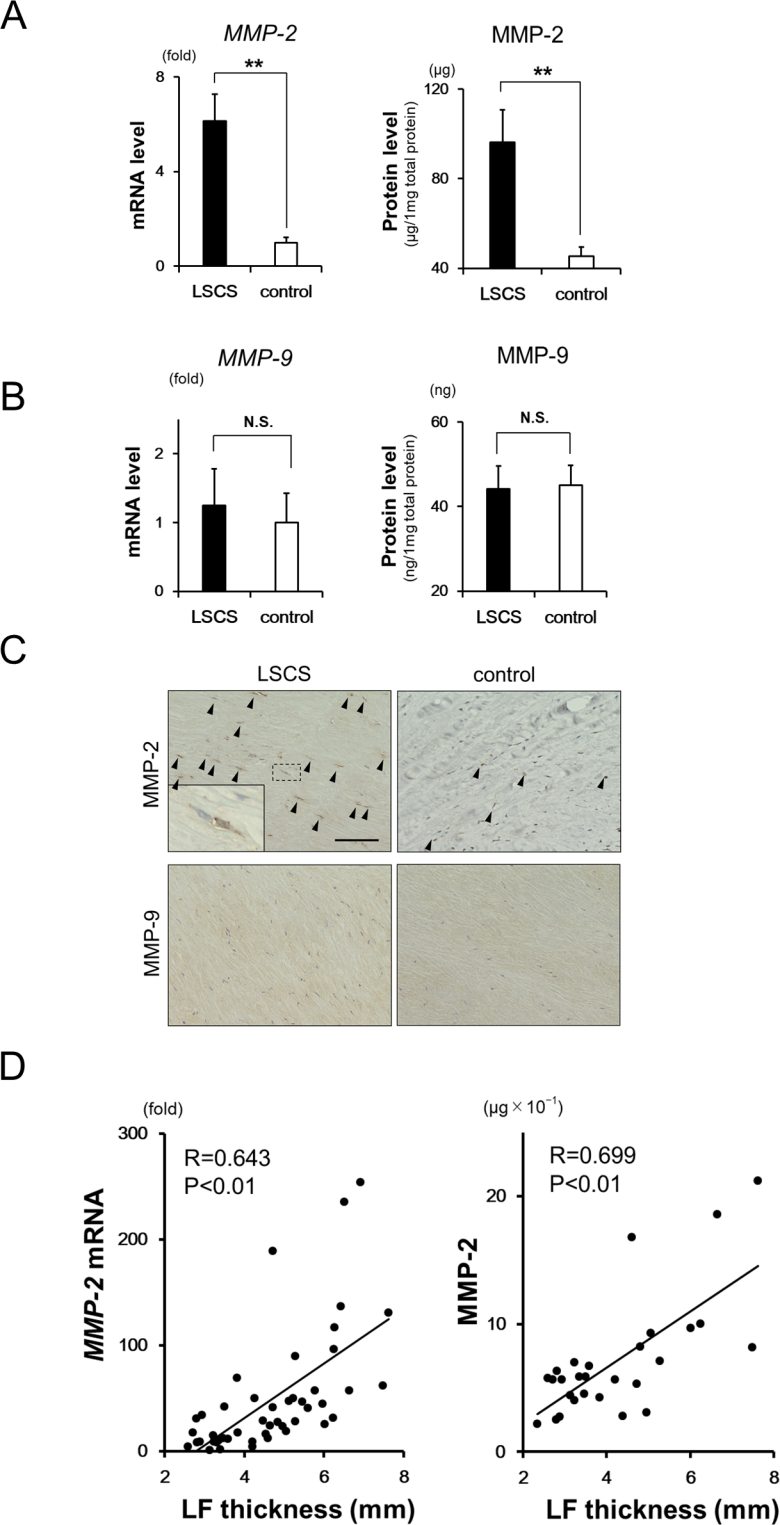
MMP-2 and -9 expressions in LF tissue. **A, B:** Comparison of MMP-2 (A) and MMP-9 (B) mRNA and protein expression in the LF of the LSCS and control groups. mRNA levels were normalized to *18S rRNA* levels. The value in the control group was set as 1. **C:** Immunohistochemical analysis of MMP-2 and -9 expressions in LF tissue from patients with LSCS (left) and control subjects (right). The inset in the left panel for MMP-2 shows a high-magnification view of the area enclosed by the dashed line. Arrowheads indicate MMP-positive cells. Scale bar, 100 μm. **D:** Correlation between MMP-2 mRNA (left) and protein (right) expression and LF thickness. Data represent the mean ± SEM (LSCS, n = 31 and control, n = 21 for mRNA assay; and n = 14 per group for protein assay). **P < 0.01 vs. control group. N.S., not significant.

MMP-2 mRNA and protein levels in LF tissue were higher in the LSCS than in the control group (Fig 2A). In contrast, MMP-9 mRNA and protein levels were similar in the two groups (Fig 2B). Accordingly, the number of MMP-2-expressing cells in LF tissue was higher in the LSCS than in the control group, whereas similarly low numbers of MMP-9-expressing cells were detected in the two groups (Fig 2C). MMP-2 mRNA and protein levels were positively correlated with LF thickness (Fig 2D). This suggests that MMP-2 is likely responsible for LF degeneration in LSCS pathogenesis, whereas MMP-9 does not exert an effect in this process.

### Relationship between loss of elastic fibers and MMP-2 expression in LF tissue

**Fig 3:**
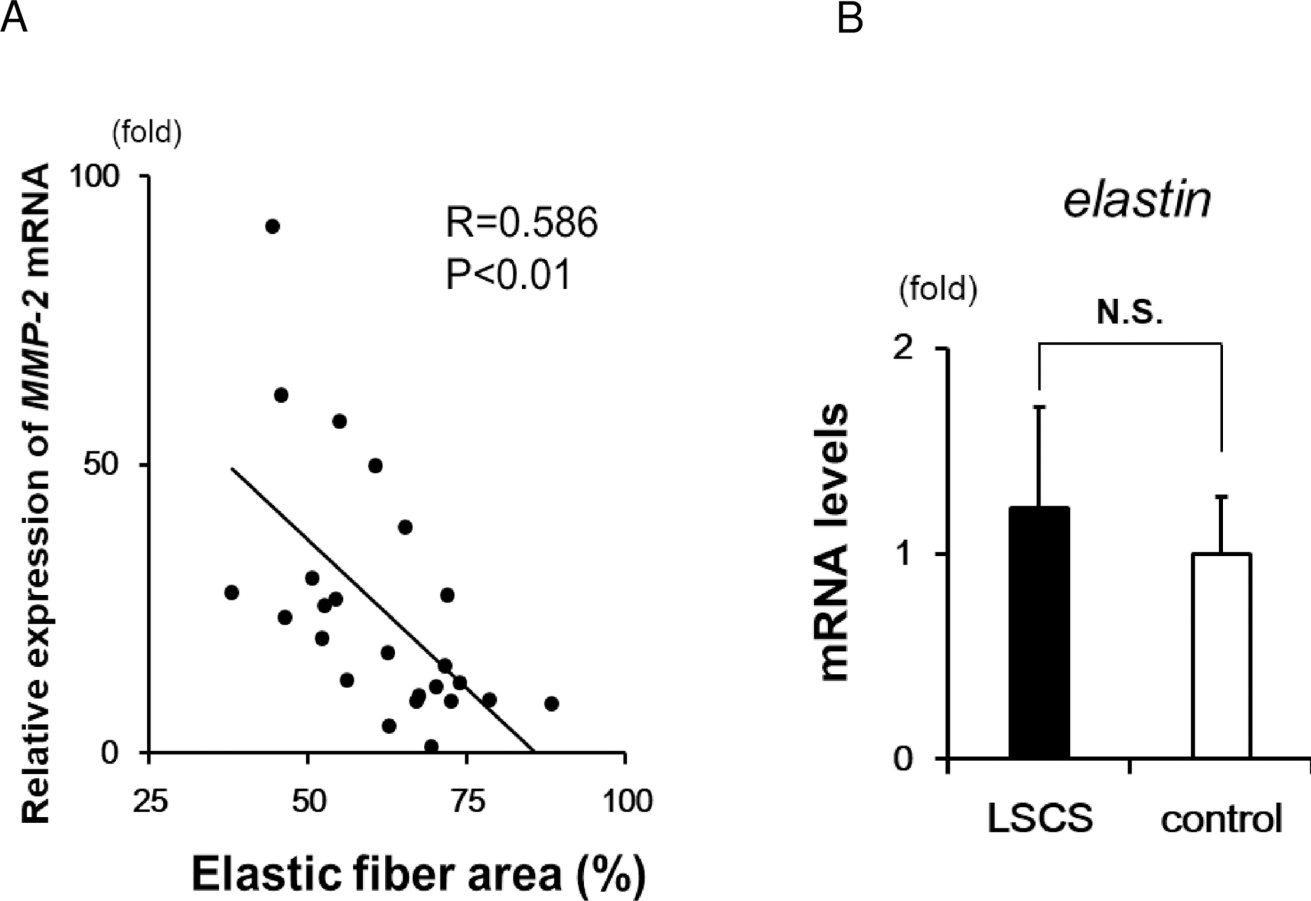
Elastic fiber assay in LF tissues. **A:** Correlation between *MMP-2* mRNA expression and elastic fiber area. The minimum value of MMP-2 expression in analyzed samples was set to 1 (LSCS, n = 13 and control, n = 11). **B:** *Elastin* mRNA expression in LF tissue (LSCS, n = 31 and control, n = 21). Data represent the mean ± SEM. N.S., not significant.

A quantitative analysis of the area occupied by elastic fibers using EVG staining revealed that the ratio of elastic fibers was negatively correlated with *MMP-2* mRNA expression (Fig 3A). In contrast, there was no difference in *elastin* expression between the LSCS and control groups, indicating similar levels of elastic fiber synthesis (Fig 3B). These findings indicate that the loss of elastic fibers in LF would be due to their degradation and not due to reduced synthesis.

### Relationship between IL-6 and MMP-2 expression in LF tissue

**Fig 4:**
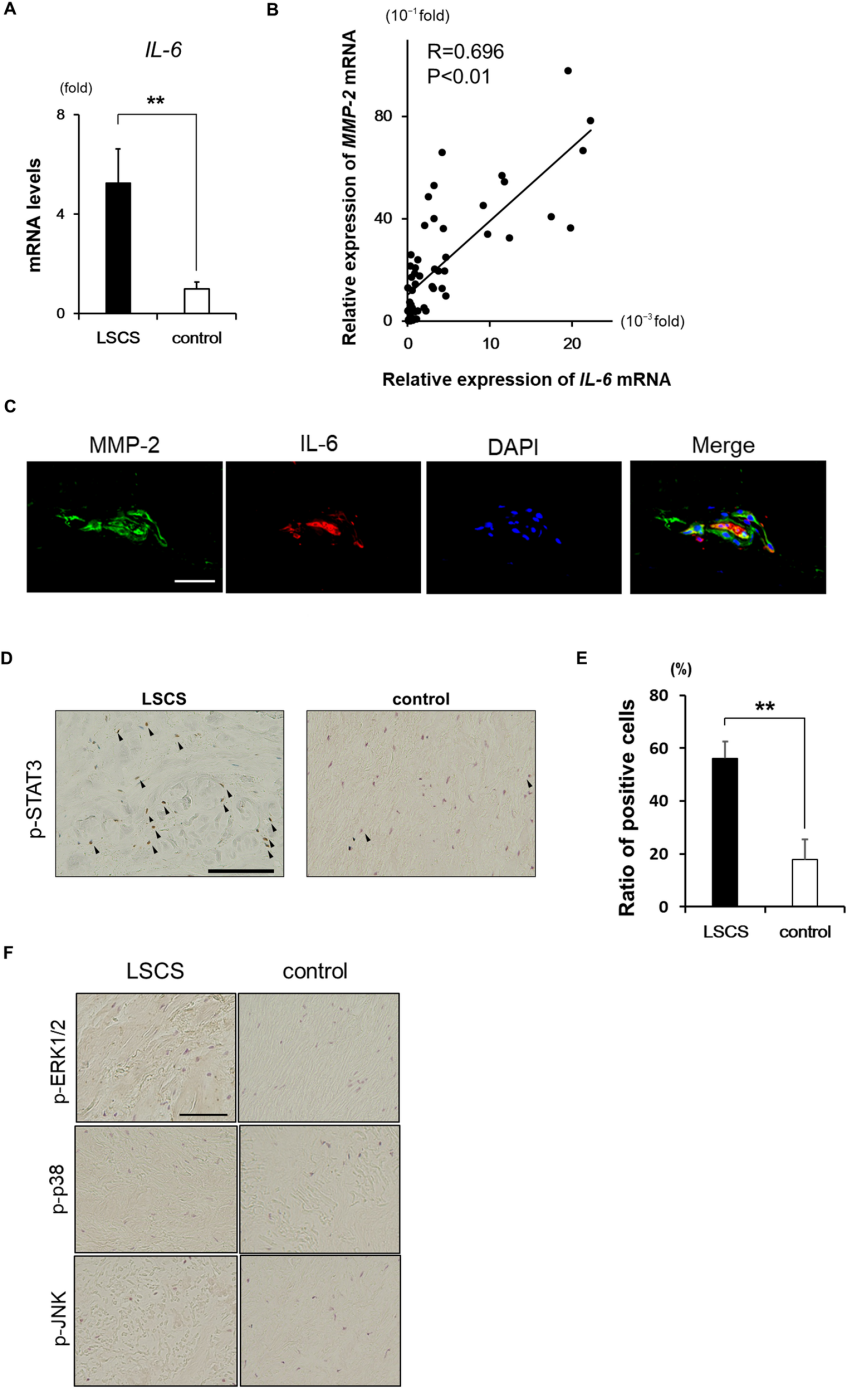
IL-6 expression in LF tissue. **A:** *IL-6* mRNA expression in LF tissues from the LSCS (n = 31) and control (n = 21) groups normalized to *18S rRNA*. Data represent the mean ± SEM. **P < 0.01 vs. control. **B:** Correlation between *IL-6* and *MMP-2* mRNA expression levels in LF tissues. **C:** Double immunofluorescence labeling of MMP-2 and IL-6. Nuclei were stained with DAPI. Scale bar, 100 μm. **D:** Immunohistochemical detection of p-STAT3. **E:** Quantitative analysis of data shown in panel D (n = 3). **F:** Immunohistochemical detection of p-ERK1/2 (upper), p-p38 (middle), and p-JNK (lower) in LF tissue from LSCS and control groups. Arrowheads indicate immunopositive cells. Scale bars, 150 μm. Regions of interest were selected from six sites (cranial, middle, and caudal sides of the dorsal and dural layers) in each sample. Images at 100× magnification were used for measurements, and the average number of p-STAT3-positive cells as a percentage of total number of cells was calculated. Data represent the mean ± SEM. **P < 0.01.

Previous reports have suggested that IL-6 derived from LF fibroblasts contributes to LF degeneration by inducing inflammation [11, 22]. Given that IL-6 has varying effects on MMP expression depending on cell type [19, 23, 24], we investigated whether IL-6 expression was correlated with MMP-2 expression in LF tissue. *IL-6* mRNA expression was higher in the LF of the LSCS as compared to the control group (Fig 4A); it was also positively correlated with *MMP-2* expression (Fig 4B). Immunohistochemical analysis revealed that MMP-2 partly co-localized with IL-6 (Fig 4C). The Janus kinase (JAK)/STAT3 and mitogen-activated protein kinase pathways are the two major pathways activated by IL-6 [25]; we therefore investigated which pathway is the main effector of the IL-6 signal in LF tissue by immunohistochemical detection of p-STAT3, p-ERK1/2, p-p38, and p-JNK. The number of cells expressing p-STAT3 was higher in the LF of the LSCS patients as compared to control subjects (Fig 4D and 4E); however, there were few cells positive for p-ERK1/2, p-p38, or p-JNK in the LF tissue samples from either group (Fig 4F). These findings indicate that LF cell-derived IL-6 may contribute to LF degeneration via activation of STAT3 and MMP-2.

### MMP-2 expression and secretion and intracellular signaling induced by IL-6 in LF fibroblasts

**Fig 5:**
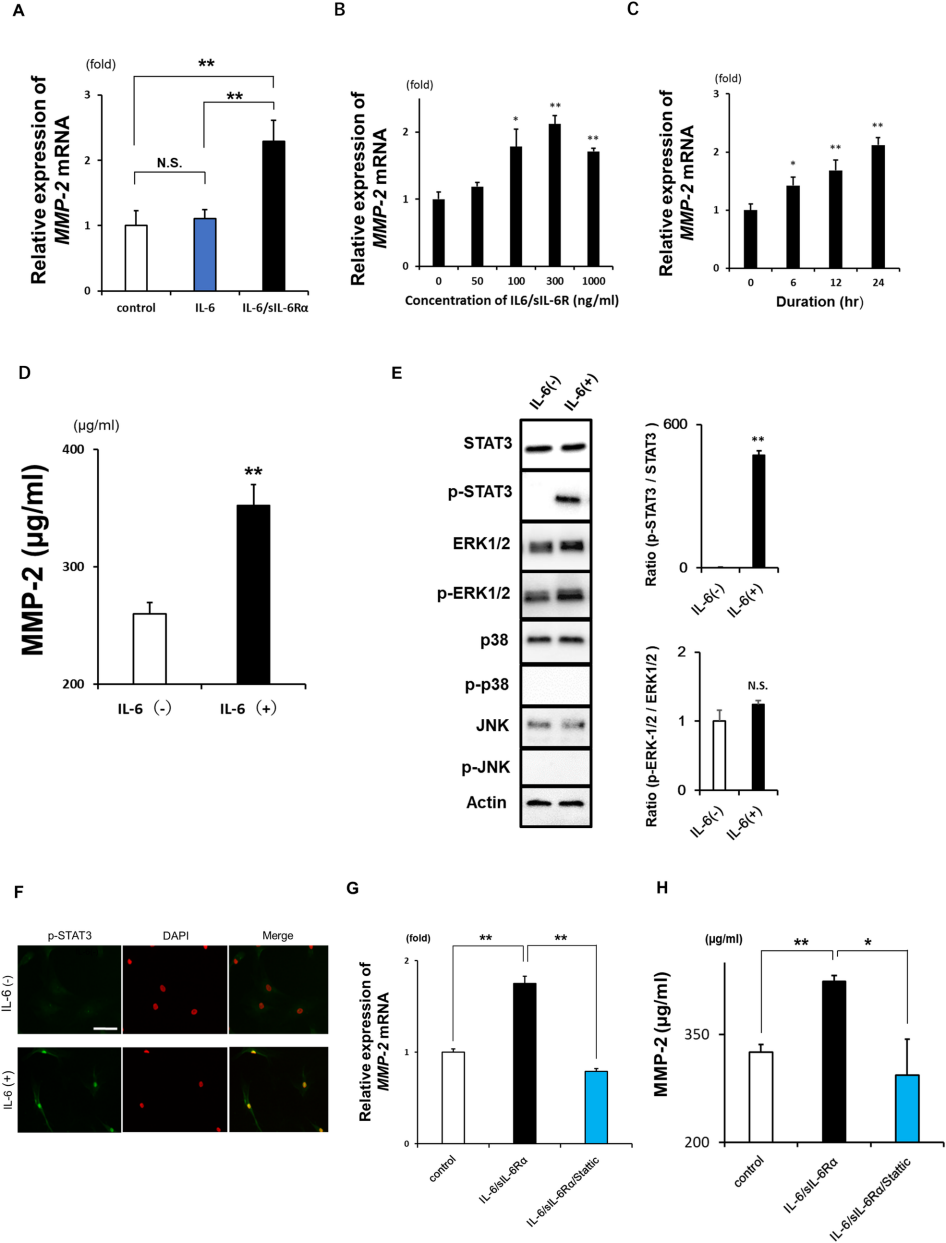
MMP-2 expression and secretion induced by IL-6 in LF fibroblasts. **A:** Changes in *MMP-2* mRNA expression in LF fibroblasts (n = 3) following administration of IL-6 protein in the presence or absence of sIL-6Rα. MMP-2 expression in LF fibroblasts without IL-6/sIL-6Rα stimulation was set as 1. **B, C:** Changes in *MMP-2* mRNA expression in LF fibroblasts (n = 3) in response to stimulation with IL-6/sIL-6Rα for 12 h at the indicated concentrations (B) and for the indicated times (C). **D:** MMP-2 protein concentration in the supernatants of culture with or without IL-6/sIL-6Rα stimulation for 24 h. **E:** Western blot analysis of STAT3, p-STAT3, ERK1/2, p-ERK1/2, p-38, p-p38, JNK, p-JNK, and actin levels in LF fibroblasts with or without IL-6/sIL-6Rα stimulation. Quantitative analysis of the ratio of p-STAT3/STAT3 (upper graph) and p-ERK1/2/ERK1/2 (lower graph) (n = 3) **F:** Nuclear translocation of p-STAT3 induced by IL-6 stimulation in LF fibroblasts. Nuclei were stained with DAPI. Scale bars, 100 μm. **G, H:** IL-6-induced *MMP-2* mRNA expression (G) and secretion (H) with or without Stattic treatment (n = 3). Data represent the mean ± SEM. *P < 0.05, **P < 0.01, N.S., not significant.

We investigated whether IL-6 directly induces MMP-2 expression and secretion in LF fibroblasts as well as the signaling mechanism involved. Treatment with IL-6 and sIL-6Rα (which formed the IL-6/sIL-6Rα complex) increased *MMP-2* mRNA expression in LF fibroblasts (Fig 5A) in a concentration- and time-dependent manner (Fig 5B and 5C). After 24 h, MMP-2 protein level in the culture medium was increased (Fig 5D). Additionally, STAT3 phosphorylation (Fig 5E) and p-STAT3 nuclear translocation (Fig 5F) were induced by IL-6/sIL-6Rα treatment, as determined by western blotting and immunocytochemistry. STAT3 inhibition by treatment with Stattic abrogated the IL-6-induced increase in MMP-2, at both the mRNA and protein levels (Fig 5G and 5H). These results demonstrate that IL-6 induces MMP-2 expression and secretion via JAK/STAT3 signaling in LF fibroblasts.

### MMP-2 activity and elastin degradation induced by IL-6 in LF fibroblasts

**Fig 6:**
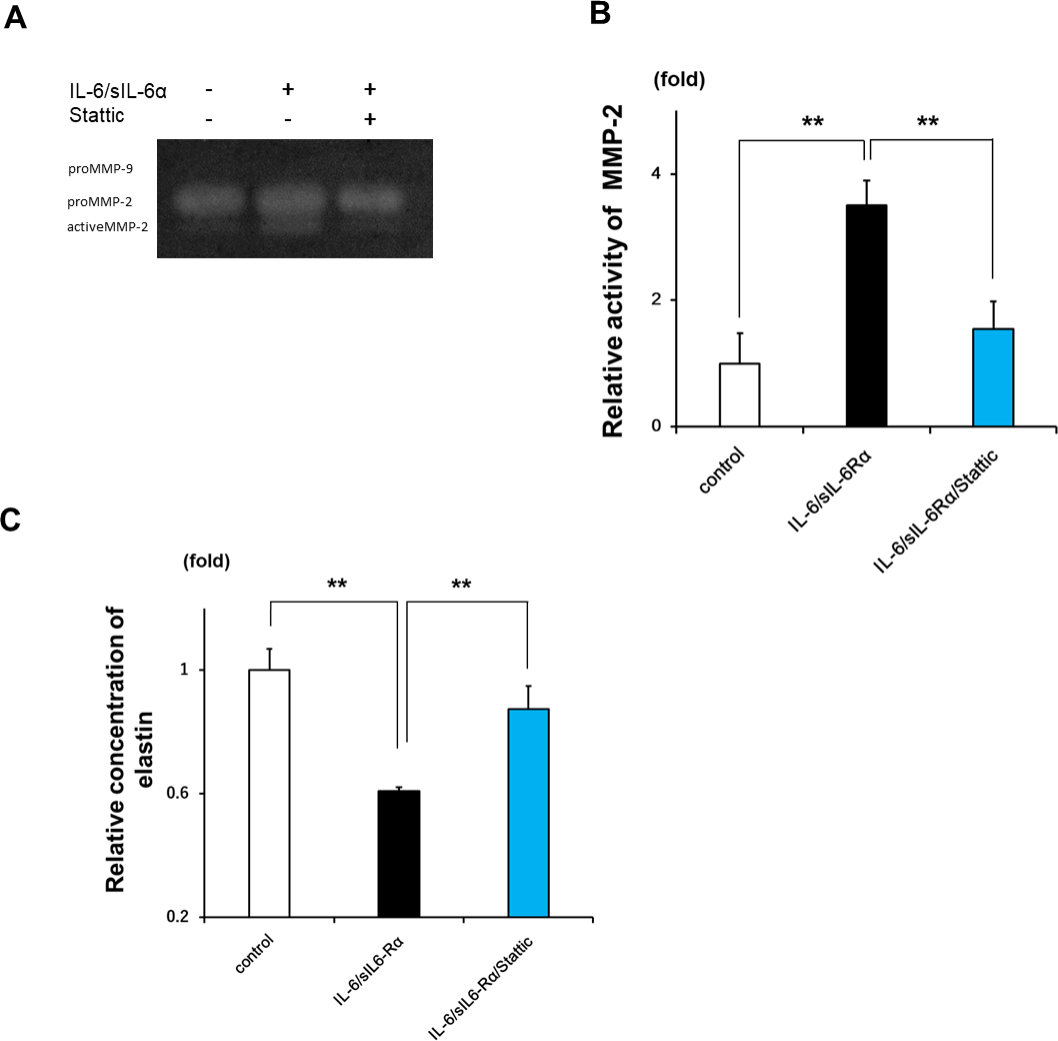
MMP-2 activation and elastolysis induced by IL-6 in LF fibroblasts. **A:** Representative gelatin zymography analysis of LF fibroblasts in response to IL-6/sIL-6Rα stimulation for 24 h with or without Stattic. **B:** Quantitative analysis of data shown in panel A (n = 3). **C:** Concentration of soluble elastin following incubation for 6 h with the supernatant of IL-6-stimulated cultures with or without Stattic (n = 3). Data represent the mean ± SEM. **P < 0.01.

To estimate MMP-2 activity, we investigated whether IL-6 could induce the conversion of proMMP-2 into active MMP-2 in LF fibroblasts. A gelatin zymography analysis of LF fibroblast culture medium revealed an increase in active MMP-2 levels in fibroblast cultures stimulated with IL-6/sIL-6Rα relative to unstimulated control samples; this effect was reversed by Stattic treatment (Fig 6A and 6B). Based on these results, we examined whether IL-6 stimulation directly influences elastin degradation. We found that soluble elastin protein concentration was decreased in the presence of IL-6, and this effect was abolished by Stattic treatment (Fig 6C). These results indicate that MMP-2 activity is induced by IL-6 in LF fibroblasts, leading to degradation of LF elastic fibers.

## Discussion

In this study, first, we confirmed that our LSCS samples exhibited severe fibrosis and elastic fiber degradation compared to the control samples. We observed that MMP-2 was more abundantly expressed in LF fibroblasts of patients with LSCS tissues than in those of control subjects, and its expression was significantly correlated with LF thickness and degree of elastic fiber loss. We found that MMP-2 expression was correlated with *IL-6* mRNA expression in LF tissues and MMP-2 partly co-localized with IL-6 in LF fibroblasts. Our *in vitro* analysis showed that MMP-2 expression, secretion, and activation in LF fibroblasts were induced by IL-6/STAT3 signaling. Additionally, elastin degradation was increased in IL-6-stimulated LF fibroblast cultures.

LF thickening is associated with symptoms such as leg pain and intermittent claudication resulting from cauda equina and nerve root compression [2]. Surgical removal of thickened LF tissue is the only curable treatment for LSCS; preventing this thickening is a more desirable therapeutic strategy, as it would obviate the need for surgical intervention. LF thickening occurs because of not only tissue hypertrophy but also LF buckling into the spinal canal due to reduced elasticity caused by loss of elastic fibers [6]. Consistent with the findings of several light and electron microscopic studies [5, 6, 15], our histological findings showed that collagen fibers were exposed and irregularly distributed and elastic fibers were scarce and disordered mainly on the dorsal side of the LSCS-LFs. The elastic system consists of oxytalan, elaunin, and elastic fibers, which are distinguished by the elastin density [20]. In our evaluation of the elastic system morphology, elaunin-like thin fibers and oxytalan-like microfibers were detected at the strongly fibrotic areas in LSCS samples, as reported by Postacchini [6]. Elaunin or oxytalan fibers, which contain microfibril bundles with fewer or no elastin deposits compared with elastic fibers, occur in areas subjected to mechanical stress [26]. The elastin degradation induced by lumbar mechanical stress could decrease the amount of elastic fibers and generate these thin fibers. It has been reported that *elastin* mRNA expression increases with aging but that elderly patients show greater loss of elastic fibers in the dorsal LF [5]. We did not observe any differences between the LSCS and control groups in terms of *elastin* expression. Thus, the disappearance of LF elastic fibers of patients with LSCS may be due to increased breakdown rather than reduced production of elastin.

MMPs are the major effectors of ECM degradation during tissue remodeling and are implicated in various pathological conditions [16]. The expression of some MMPs has been found to be elevated in fibroblasts of hypertrophied LF tissue and is thought to affect LF degeneration [14]. Gelatinases, including MMP-2 and -9, can degrade elastin molecules and are upregulated in diseases of reduced elasticity, including abdominal aortic aneurysm and pulmonary fibrosis [17, 18]. In LSCS, increased activation of MMPs, especially of MMP-2, in LF fibroblasts could lead to the degradation of elastic fibers in LFs, thereby resulting in decreased elasticity.

IL-6 modulates MMP expression and activity [23, 24]. IL-6 activates JAK and STAT proteins; the latter is translocated to the nucleus where it activates target gene transcription [13, 25]. The JAK1/STAT3 pathway plays an important role in the inflammatory response in LSCS, primarily in the dorsal LF [21]. STAT3 is a transcription factor that induces MMP-2 expression and is thought to directly bind to the *MMP-2* promoter in human neoplastic cells [27]. We therefore speculated that IL-6 contributes to the degradation of elastic fibers through induction of MMP-2 via JAK/STAT3 signaling in LSCS. Indeed, we found that IL-6 activated STAT3, which was translocated to the nucleus and induced MMP-2 expression in LF fibroblasts. It has been suggested that mechanical stress causes inflammation and LF tissue degeneration [5, 7–9]. We previously reported that IL-6 is abundantly expressed in LF tissue in patients with LSCS, and its expression is induced by mechanical stress-promoted Angptl2 in LF fibroblasts [10, 11]. IL-6 also stimulates collagen expression in LF cells [28]. Thus, mechanical stress-induced IL-6 may be responsible for irreversible pathological LF remodeling, including fibrosis and loss of elastic fibers.

MMP-2 has angiogenic activity [29]; increased angiogenesis has been detected in the area of collagen accumulation in severely hypertrophied LF and is responsible for LF fibrosis [30]. Thus we speculate that MMP-2 contributes not only to the loss of elastic fibers leading to a decrease in elasticity, but also to LF neovascularization and fibrosis.

MMPs are initially secreted as inactive proMMPs and are activated by other MMPs [31, 32]. This process is suppressed by tissue inhibitor of metalloproteinases (TIMPs). MMP-2 is mainly activated by the balance between TIMP-2 and MMP-14, which is a membrane type 1 MMP [16, 31, 32]. Inactive zymogen proMMP-2 forms a complex with homodimerized MMP-14 and TIMP-2 at the cell surface; this interaction is necessary for the conversion of proMMP-2 into active MMP-2. Dysregulation of this balance, for example, through TIMP-2 overexpression, blocks MMP-2 activation [31, 32]. In this study, we found that MMP-14 and TIMP-2 were both expressed at higher levels in patients with LSCS than in control subjects, corresponding to a higher ratio of MMP-14 to TIMP-2 in degenerative LFs (data not shown). This modulation of the expression of the components involved in MMP conversion has been observed in intervertebral disc degeneration and giant-cell arteritis [33, 34]. MMP-14 expression is regulated by many growth factors, mediators, and signaling pathways; in atherosclerotic plaques, IL-6 has been shown to enhance MMP-14 expression [32, 35]. Thus, MMP-2 activation in the LF could be increased under conditions of inflammation in LSCS patients. Our in vitro experiment also found that IL-6 stimulation induced MMP-2 activation accompanied by MMP-2 overexpression, but the detailed mechanism remains unclear. Further investigation of MMP-2 activation in LF fibroblasts is necessary for elucidating the elastin degradation.

There were several limitations of our study. First, the mean age of patients with LSCS was higher than that of control patients. It was previously reported that qualitative changes in LF associated with aging are due to mechanical loading [5]; thus, we consider that the degradation of elastic fibers is not a normal part of aging but a pathological process. Second, our control group was small and we were unable to obtain all of the samples by *en bloc* resection because minimally invasive surgery has been adopted as LDH treatment at our institution. We therefore had to rely on the histopathological examination of LF in control samples within the possible range.

In conclusion, we demonstrated that MMP-2 is highly expressed in the LF tissue of patients with LSCS, and that MMP-2, induced by IL-6 in LF fibroblasts, promotes the loss of elastic fibers during LF tissue degeneration. These findings indicate that MMP-2 is a key therapeutic target for the preventive treatment of LSCS.
